# Bounds for the norm of lower triangular matrices on the Cesàro weighted sequence space

**DOI:** 10.1186/s13660-017-1339-6

**Published:** 2017-04-04

**Authors:** Davoud Foroutannia, Hadi Roopaei

**Affiliations:** Department of Mathematics, Vali-e-Asr University of Rafsanjan, Rafsanjan, Iran

**Keywords:** norm, lower triangular matrix, Nörlund matrix, weighted mean matrix, weighted sequence space

## Abstract

This paper is concerned with the problem of finding bounds for the norm of lower triangular matrix operators from $l_{p}(w)$ into $c_{p} (w)$, where $c_{p}(w)$ is the Cesàro weighted sequence space and $(w_{n})$ is a non-negative sequence. Also this problem is considered for lower triangular matrix operators from $c_{p}(w)$ into $l_{p} (w)$, and the norms of certain matrix operators such as Cesàro, Nörlund and weighted mean are computed.

## Introduction

Let $p\ge1$ and *ω* denote the set of all real-valued sequences. The space $l_{p}$ is the set of all real sequences $x=(x_{n})\in\omega$ such that

$$\Vert x \Vert _{p}= \Biggl(\sum_{n=1}^{\infty} \vert x_{n} \vert ^{p} \Biggr)^{1/p}< \infty. $$

If $w=(w_{n})\in\omega$ is a non-negative sequence, we define the weighted sequence space $l_{p}(w)$ as follows:

$$l_{p}(w):= \Biggl\{ x=(x_{n})\in\omega : \sum _{n=1}^{\infty}w_{n} \vert x_{n} \vert ^{p}< \infty \Biggr\} , $$

with norm $\Vert \cdot \Vert _{p,w}$, which is defined in the following way:

$$\Vert x \Vert _{p,w}= \Biggl( \sum_{n=1}^{\infty}w_{n} \vert x_{n} \vert ^{p} \Biggr)^{1/p}. $$

Let $C=(c_{n,k})$ denote the Cesàro matrix. We recall that the elements $c_{n,k}$ of the matrix *C* are given by

$$\begin{aligned} c_{n,k}= \textstyle\begin{cases} \frac{1}{n} & \mbox{for }1\leq k \leq n,\\ 0 & \mbox{for }k>n. \end{cases}\displaystyle \end{aligned}$$

The sequence space defined by

$$\begin{aligned} c_{p}(w) =& \bigl\{ (x_{n})\in\omega : Cx\in l_{p}(w) \bigr\} \\ =& \Biggl\{ (x_{n})\in\omega : \sum_{n=1}^{\infty}w_{n} \Biggl\vert \frac{1}{n}\sum_{i=1}^{n}x_{i} \Biggr\vert ^{p}< \infty \Biggr\} \end{aligned}$$

is called the Cesàro weighted sequence space, and the norm $\Vert \cdot \Vert _{p,w,c}$ of the space is defined by

$$\Vert x \Vert _{p,w,c}= \Biggl( \sum_{n=1}^{\infty}w_{n} \Biggl\vert \frac{1}{n}\sum_{i=1}^{n}x_{i} \Biggr\vert ^{p} \Biggr)^{1/p}. $$

The Cesàro sequence spaces were studied in [1], where $w_{n}=1$ for all *n*. It is significant that in the special case $w_{n}=1$, we have $l_{p}(w)=l_{p}$ and $c_{p}(w)=c_{p}$.

Let $(w_{n})$ be a non-negative sequence and $A=(a_{n,k})$ be a lower triangular matrix with non-negative entries. In this paper, we shall consider the inequality of the form

$$\Vert Ax \Vert _{p,w,c}\le U \Vert x \Vert _{p,w}, $$

and the inequality of the form

$$\Vert Ax \Vert _{p,w}\le U \Vert x \Vert _{p,w,c}, $$

where $x=(x_{n})$ is a non-negative sequence. The constant *U* does not depend on *x*, and we seek the smallest possible value of *U*. We write $\Vert A \Vert _{p,w,c}$ for the norm of *A* as an operator from $l_{p}(w)$ into $c_{p}(w)$, $\Vert A \Vert _{p,c}$ for the norm of *A* as an operator from $l_{p}$ into $c_{p}$, $\Vert A \Vert _{c,p,w}$ for the norm of *A* as an operator from $c_{p}(w)$ into $l_{p}(w)$, $\Vert A \Vert _{c,p}$ for the norm of *A* as an operator from $c_{p}$ into $l_{p}$, $\Vert A \Vert _{p,w}$ for the norm of *A* as an operator from $l_{p}(w)$ into itself and $\Vert A \Vert _{p}$ for the norm of *A* as an operator from $l_{p}$ into itself.

The problem of finding the norm of a lower triangular matrix on the sequence spaces $l_{p}$ and $l_{p}(w)$ has been studied before in [2–8]. In the study, we will expand this problem for matrix operators from $l_{p}(w)$ into $c_{p} (w)$ and matrix operators from $c_{p}(w)$ into $l_{p} (w)$, and we consider certain matrix operators such as Cesàro, Nörlund and weighted mean. The study is an extension of some results obtained by [3, 7].

## The norm of matrix operators from $l_{p}(w)$ into $c_{p}(w)$

In this section, we tend to compute the bounds for the norm of lower triangular matrix operators from $l_{p}(w)$ into $c_{p} (w)$. In particular, we apply our results for lower triangular matrix operators from $l_{p}$ into $c_{p}$, when $w_{n}=1$ for all *n*.

Throughout this paper, let $A=(a_{n,k})$ be a matrix with non-negative real entries i.e., $a_{n,k}\ge0$, for all *n*, *k*. This implies that $\Vert Ax \Vert _{p,w,c}\le \Vert A \vert x \vert \Vert _{p,w,c}$, and hence the non-negative sequences are sufficient to determine the norm of *A*. We say that $A=(a_{n,k})$ is lower triangular if $a_{n,k}=0$ for $n< k$. A non-negative lower triangular matrix is called a summability matrix if $\sum_{k=1}^{n}a_{n,k}=1$ for all *n*.

We first state some lemmas from [3, 7], which are needed for our main result. Set $\xi^{+}=\max(\xi,0)$ and $\xi^{-}=\min(\xi,0)$ and $p^{*}=p/(p-1)$.

### Lemma 2.1

[3], Lemma 2.1

*Let* *a* *and* *x* *be two non*-*negative sequences*, *then for all* *n*,

$$\sum_{k=1}^{n}a_{k}x_{k} \leq \Biggl\{ \max_{1\le k\le n}\frac{1}{n-k+1}\sum _{j=k}^{n}x_{j} \Biggr\} \sum _{k=1}^{n}(n-k+1) (a_{k}-a_{k-1})^{+}. $$

### Lemma 2.2

[3], Lemma 2.2

*Let* $N\geq1$, *and let* *a* *and* *x* *be two non*-*negative sequences*. *If* $x_{N}\geq x_{N+1}\geq\cdots\geq0$ *and* $x_{n}=0$ *for* $n< N$, *then*

$$\sum_{k=1}^{n}a_{k}x_{k} \geq \Biggl(\frac{1}{n}\sum_{j=1}^{n}x_{j} \Biggr) \Biggl\{ na_{N}+\frac{n}{n-N+1}\sum _{k=N+1}^{n}(n-k+1) (a_{k}-a_{k-1})^{-} \Biggr\} $$

*for all* *n*.

### Lemma 2.3

[7], Lemma 1.4

*Let* $p>1$ *and* $w=(w_{n})$ *be a decreasing sequence with non*-*negative entries and* $\sum_{n=1}^{\infty}\frac{w_{n}}{n}$ *be divergent*. *Let* $N\geq1$ *and the matrix* $C_{N}=(c_{n,k}^{N})$ *be with the following entries*:

$$\begin{aligned} c_{n,k}^{N}= \textstyle\begin{cases} \frac{1}{n+N-1} & \textit{for }n\geq k, \\ 0 & \textit{for }n< k. \end{cases}\displaystyle \end{aligned}$$

*Then* $\Vert C_{N} \Vert _{p,w}$ *is determined by non*-*negative decreasing sequences and* $\Vert C_{N} \Vert _{p,w}=p^{*}$.

Note that $C_{1}$ is the well-known Cesàro matrix.

### Lemma 2.4

[7], Lemma 1.5

*If* $p>1$ *and* *x* *and* *w* *are two non*-*negative sequences and also* *w* *is decreasing*, *then*

$$\sum_{j=1}^{\infty}w_{j}\max _{1\le i\le j} \Biggl(\frac{1}{j-i+1}\sum _{k=i}^{j}x_{k} \Biggr)^{p}\le \bigl(p^{*}\bigr)^{p}\sum_{k=1}^{\infty}w_{k}x_{k}^{p}. $$

We set $a_{0,0}=0$ and $a_{n,0}=0$ for $n\geq1$ and

$$\begin{aligned} &M_{A}=\sup_{n\geq1} \Biggl\{ \sum _{k=1}^{n}\frac{n-k+1}{n} \Biggl(\sum _{i=k}^{n}a_{i,k}-\sum _{i=k-1}^{n}a_{i,k-1} \Biggr)^{+} \Biggr\} , \\ &m_{A}=\sup_{N\geq1}\inf_{n\geq N} \Biggl\{ \sum_{i=N}^{n}a_{i,N}+ \frac{1}{n-N+1} \sum_{k=N+1}^{n}(n-k+1) \Biggl(\sum_{i=k}^{n}a_{i,k}-\sum _{i=k-1}^{n}a_{i,k-1} \Biggr)^{-} \Biggr\} . \end{aligned}$$

We are now ready to present the main result of this section.

### Theorem 2.5

*Suppose that* $p>1$ *and* $w=(w_{n})$ *is a decreasing sequence with non*-*negative entries*. *If* $A=(a_{n,k})$ *is a lower triangular matrix with non*-*negative entries*, *then we have the following statements*.

(i) $\Vert A \Vert _{p,w,c}\leq p^{*}M_{A}$. *Moreover*, *if* $M_{A}<\infty$, *then* *A* *is a bounded matrix operator from* $l_{p}(w)$ *into* $c_{p}(w)$.
(ii) *If* $\sum_{n=1}^{\infty}\frac{w_{n}}{n}$ *is divergent and* $(\frac{w_{n}}{w_{n+1}})$ *is decreasing*, *then* $\Vert A \Vert _{p,w,c}\geq p^{*}m_{A}$.

*Therefore if* $w=(w_{n})$ *is a decreasing sequence with non*-*negative entries and* $(\frac{w_{n}}{w_{n+1}})$ *is decreasing and* $\sum_{n=1}^{\infty}\frac{w_{n}}{n}=\infty$, *then*

$$p^{*}m_{A}\leq \Vert A \Vert _{p,w,c}\leq p^{*}M_{A}. $$

*In particular*, *if* $w_{n}=1$ *for all* *n* *and if* $M_{A}<\infty$, *then* *A* *is a bounded matrix operator from* $l_{p}$ *into* $c_{p}$ *and* $p^{*}m_{A}\leq \Vert A \Vert _{p,c}\leq p^{*}M_{A}$.

### Proof

(i) Let $(x_{n})$ be a non-negative sequence. By using Lemma 2.1, we get

$$\begin{aligned} &\sum_{k=1}^{n} \Biggl(\frac{1}{n}\sum _{i=k}^{n}a_{i,k} \Biggr)x_{k}\\ &\quad\leq\Biggl\{ \max_{1\le k\le n}\frac{1}{n-k+1} \sum_{j=k}^{n}x_{j} \Biggr\} \sum _{k=1}^{n}\frac {n-k+1}{n} \Biggl(\sum _{i=k}^{n}a_{i,k}-\sum _{i=k-1}^{n}a_{i,k-1} \Biggr)^{+} \\ &\quad \leq M_{A}\max_{1\le k\le n} \Biggl\{ \frac{1}{n-k+1}\sum _{j=k}^{n}x_{j} \Biggr\} . \end{aligned}$$

By applying Lemma 2.4, we deduce that

$$\begin{aligned} \sum_{n=1}^{\infty}w_{n} \Biggl(\sum _{k=1}^{n} \Biggl(\frac{1}{n}\sum _{i=k}^{n}a_{i,k} \Biggr)x_{k} \Biggr)^{p} \leq&M_{A}^{p} \sum_{n=1}^{\infty} w_{n}\max _{1\leq k\le n} \Biggl(\frac{1}{n-k+1}\sum _{j=k}^{n}x_{j} \Biggr)^{p} \\ \leq&\bigl(p^{*}M_{A}\bigr)^{p}\sum _{k=1}^{\infty}w_{k}x_{k}^{p}. \end{aligned}$$

(ii) We have $m_{A}=\sup_{N\geq1}\beta_{N}$, where

$$\beta_{N}=\inf_{n\geq N} \Biggl\{ \sum _{i=N}^{n}a_{i,N}+\frac{1}{n-N+1} \sum _{k=N+1}^{n}(n-k+1) \Biggl(\sum _{i=k}^{n}a_{i,k}-\sum _{i=k-1}^{n}a_{i,k-1} \Biggr)^{-} \Biggr\} . $$

Let $N\geq1$, so that $\beta_{N}\geq0$. If $y=(y_{n})$ is a decreasing sequence with non-negative entries and $\Vert y \Vert _{p,w}=1$, we set $x_{1}=x_{2}=\cdots=x_{N-1}=0$ and

$$x_{n+N-1}=\biggl(\frac{w_{n}}{w_{n+N-1}}\biggr)^{1/p}y_{n} $$

for all $n\geq1$. So $\Vert x \Vert _{p,w}= \Vert y \Vert _{p,w}=1$, and from Lemma 2.2 it follows that

$$\begin{aligned} \Vert A \Vert _{p,w,c}^{p}&\geq\sum _{n=1}^{\infty}w_{n} \Biggl(\sum _{k=1}^{n} \Biggl(\frac{1}{n}\sum _{i=k}^{n}a_{i,k} \Biggr)x_{k} \Biggr)^{p} \\ &\geq \beta_{N}^{p}\sum_{n=1}^{\infty}w_{n} \Biggl(\frac{1}{n}\sum_{j=1}^{n}x_{j} \Biggr)^{p} \\ &=\beta_{N}^{p}\sum_{n=1}^{\infty}w_{n+N-1} \Biggl(\frac{1}{n+N-1}\sum_{j=1}^{n}x_{j+N-1} \Biggr)^{p} \\ &=\beta_{N}^{p}\sum_{n=1}^{\infty}w_{n+N-1} \Biggl(\frac{1}{n+N-1}\sum_{j=1}^{n} \biggl(\frac{w_{j}}{w_{j+N-1}}\biggr)^{1/p}y_{j} \Biggr)^{p} \\ &\geq\beta_{N}^{p} \Vert C_{N}y \Vert _{p,w,}^{p}. \end{aligned}$$

By Lemma 2.3, we conclude that $\Vert A \Vert _{p,w,c}\geq p^{*}\beta_{N}$, so

$$\Vert A \Vert _{p,w,c}\geq p^{*}m_{A}. $$

 □

In what follows we assume that $w=(w_{n})$ is a decreasing sequence with non-negative entries and $(\frac{w_{n}}{w_{n+1}})$ is decreasing and $\sum_{n=1}^{\infty}\frac{w_{n}}{n}=\infty$.

At first we bring a corollary of Theorem 2.5 for a lower triangular matrix $A=(a_{n,k})$. The rows of $C_{1}A$ are increasing, where $C_{1}$ is the Cesàro matrix and

$$\begin{aligned} (C_{1}A)_{n,k}=\sum_{i=1}^{\infty}c^{1}_{n,i}a_{i,k}= \frac{1}{n}\sum_{i=k}^{n}a_{i,k},\quad (n, k=1,2,\ldots). \end{aligned}$$

### Corollary 2.6

*Suppose that* $p>1$ *and* $A=(a_{n,k})$ *is a non*-*negative lower triangular matrix that* $\sum_{i=k-1}^{n}a_{i,k-1}\le \sum_{i=k}^{n}a_{i,k}$ *for* $1< k \leq n$. *Then*

$$\Vert A \Vert _{p,w,c}=p^{*}\sup_{n\geq1} a_{n,n}. $$

*In particular*, $\Vert I \Vert _{p,w,c}=p^{*}$, *where* *I* *is the identity matrix*.

### Proof

Since the finite sequence $(\sum_{i=k}^{n}a_{i,k} )_{k=1}^{n}$ is increasing for each *n*, we have

$$\begin{aligned} \Biggl(\sum_{i=k}^{n}a_{i,k}-\sum _{i=k-1}^{n}a_{i,k-1} \Biggr)^{+} =\sum_{i=k}^{n}a_{i,k}- \sum_{i=k-1}^{n}a_{i,k-1} \end{aligned}$$

for $1\leq k \le n$. Hence

$$\begin{aligned} M_{A} =&\sup_{n\geq1} \Biggl\{ \sum _{k=1}^{n}\frac{n-k+1}{n} \Biggl(\sum _{i=k}^{n}a_{i,k}-\sum _{i=k-1}^{n}a_{i,k-1} \Biggr) \Biggr\} \\ =&\sup_{n\geq1}\frac{1}{n}\sum _{k=1}^{n}\sum_{i=k}^{n}a_{i,k} \le\sup_{n\geq1} a_{n,n}. \end{aligned}$$

Moreover,

$$\begin{aligned} \Biggl(\sum_{i=k}^{n}a_{i,k}-\sum _{i=k-1}^{n}a_{i,k-1} \Biggr)^{-}=0\quad (1\leq k \le n) \end{aligned}$$

and

$$\begin{aligned} m_{A}=\sup_{N\geq1}\inf_{n\geq N}\sum _{i=N}^{n}a_{i,N}=\sup _{n\geq1} a_{n,n}. \end{aligned}$$

Hence, according to Theorem 2.5, we obtain the desired result. □

### Example 2.7

Let $A=(a_{n,k})$ be defined by

$$\begin{aligned} a_{n,k}= \textstyle\begin{cases} \frac{1}{n^{2}} & \mbox{for } k< n, \\ \frac{2n-1}{n} & \mbox{for } k=n, \\ 0 & \mbox{for } k>n. \end{cases}\displaystyle \end{aligned}$$

Since the finite sequence $(\sum_{i=k}^{n}a_{i,k} )_{k=1}^{n}$ is increasing for each *n* and $\sup_{n\geq1} a_{n,n}=2$, by Corollary 2.6, we have $\Vert A \Vert _{p,w,c}=2p^{*}$.

Now, in the second case, we state some corollaries of Theorem 2.5 for a lower triangular matrix *A*, where the rows of $C_{1}A$ are decreasing.

### Corollary 2.8

*Suppose that* $p>1$ *and* $A=(a_{n,k})$ *is a lower triangular matrix with* $\sum_{i=k-1}^{n}a_{i,k-1}\ge\sum_{i=k}^{n}a_{i,k}$ *for* $1< k \leq n$. *Then*

$$p^{*} \Biggl(\inf_{n\geq 1}\frac{1}{n}\sum _{k=1}^{n}\sum_{i=k}^{n}a_{i,k} \Biggr)\leq \Vert A \Vert _{p,w,c}\leq p^{*} \Biggl(\sup _{n\geq1}\sum_{i=1}^{n}a_{i,1} \Biggr). $$

*In particular*, *for summability matrices the left*-*hand side of the above inequality reduces to* $p^{*}$.

*Moreover*, *if the right*-*hand side of the above inequality is finite*, *then* *A* *is a bounded matrix operator from* $l_{p}(w)$ *into* $c_{p}(w)$.

### Proof

Since the finite sequence $(\sum_{i=k}^{n}a_{i,k} )_{k=1}^{n}$ is decreasing for each *n*, we have

$$\begin{aligned} \Biggl(\sum_{i=k}^{n}a_{i,k}-\sum _{i=k-1}^{n}a_{i,k-1} \Biggr)^{+}=0\quad (1< k \le n), \end{aligned}$$

and $(\sum_{i=1}^{n}a_{i,1}-\sum_{i=0}^{n}a_{i,0} )^{+} =\sum_{i=1}^{n}a_{i,1}$. Hence $M_{A}=\sup_{n\geq1}\sum_{i=1}^{n}a_{i,1}$. Moreover,

$$\begin{aligned} \Biggl(\sum_{i=k}^{n}a_{i,k}-\sum _{i=k-1}^{n}a_{i,k-1} \Biggr)^{-} =\sum_{i=k}^{n}a_{i,k}- \sum_{i=k-1}^{n}a_{i,k-1}, \end{aligned}$$

for $1< k \leq n$, so

$$\begin{aligned} m_{A} =&\sup_{N\geq1}\inf_{n\geq N} \Biggl\{ \sum_{i=N}^{n}a_{i,N}+ \frac{1}{n-N+1} \sum_{k=N+1}^{n}(n-k+1) \Biggl(\sum_{i=k}^{n}a_{i,k}-\sum _{i=k-1}^{n}a_{i,k-1} \Biggr) \Biggr\} \\ =&\sup_{N\geq1}\inf_{n\geq N}\frac{1}{n-N+1}\sum _{k=N}^{n}\sum_{i=k}^{n}a_{i,k} \\ \ge&\inf_{n\geq1}\frac{1}{n}\sum _{k=1}^{n}\sum_{i=k}^{n}a_{i,k}. \end{aligned}$$

Therefore, by Theorem 2.5, we prove the desired result. □

The two examples of Corollary 2.8 are given as follows.

### Example 2.9

Suppose that $\alpha\ge2$ and the matrix $A=(a_{n,k})$ is defined by

$$\begin{aligned} a_{n,k}= \textstyle\begin{cases} \frac{1}{n^{\alpha}} & \mbox{for } n\geq k, \\ 0 & \mbox{for } n< k. \end{cases}\displaystyle \end{aligned}$$

Since $\sum_{i=k}^{n}a_{i,k}=\sum_{i=k}^{n} \frac{1}{i^{\alpha}}$ and $\sum_{i=k-1}^{n}a_{i,k-1}\ge\sum_{i=k}^{n}a_{i,k}$ for $1< k \leq n$, we have $0\le \Vert A \Vert _{p,w,c}\leq p^{*}\zeta(\alpha)$, where $\zeta(\alpha )=\sum_{n=1}^{\infty}\frac{1}{n^{\alpha}}$.

### Example 2.10

Suppose that the matrix $A=(a_{n,k})$ is defined by

$$\begin{aligned} a_{n,k}= \textstyle\begin{cases} \frac{1}{n(n+1)} & \mbox{for }n\geq k, \\ 0 & \mbox{for } n< k. \end{cases}\displaystyle \end{aligned}$$

Since $\sum_{i=k}^{n}a_{i,k}=\sum_{i=k}^{n}\frac{1}{i(i+1)}$, by Corollary 2.8, we have $0\le \Vert A \Vert _{p,w,c}\leq p^{*}$.

We apply the above corollary to the following two special cases.

Let $(a_{n})$ be a non-negative sequence with $a_{1}>0$, and $A_{n}=a_{1}+\cdots+a_{n}$. The Nörlund matrix $N_{a}=(a_{n,k})$ is defined as follows:

$$\begin{aligned} a_{n,k}= \textstyle\begin{cases} \frac{a_{n-k+1}}{A_{n}}& \mbox{for }1\le k\le n,\\ 0& \mbox{for } k>n. \end{cases}\displaystyle \end{aligned}$$

Also the weighted mean matrix $M_{a}=(a_{n,k})$ is defined by

$$\begin{aligned} a_{n,k}= \textstyle\begin{cases} \frac{a_{k}}{A_{n}}& \mbox{for }1\le k\le n,\\ 0& \mbox{for } k>n. \end{cases}\displaystyle \end{aligned}$$

### Corollary 2.11

*Suppose that* $p>1$ *and* $N_{a}=(a_{n,k})$ *is the Nörlund matrix and* $(a_{n})$ *is an increasing sequence*. *Then*

$$p^{*}\leq \Vert N_{a} \Vert _{p,w,c}\leq p^{*} \Biggl(\sup _{n\geq1}\sum_{i=1}^{n} \frac{a_{i}}{A_{i}} \Biggr). $$

### Proof

Since $N_{a}$ is a summability matrix and $\sum_{i=1}^{n}a_{i,1}=\sum_{i=1}^{n}\frac{a_{i}}{A_{i}}$, by applying Corollary 2.8, we have the desired result. □

### Corollary 2.12

*Suppose that* $p>1$ *and* $M_{a}=(a_{n,k})$ *is the weighted mean matrix and* $(a_{n})$ *is a decreasing sequence*. *Then*

$$p^{*}\leq \Vert M_{a} \Vert _{p,w,c}\leq p^{*}a_{1} \Biggl(\sup_{n\geq1}\sum_{i=1}^{n} \frac{1}{A_{i}} \Biggr). $$

### Proof

Since $M_{a}$ is a summability matrix and $\sum_{i=1}^{n}a_{i,1}=\sum_{i=1}^{n}\frac{a_{1}}{A_{i}}$, by Corollary 2.8, the proof is obvious. □

Finally, in the third case, if the rows of $C_{1}A$ are neither increasing nor decreasing, we present the following theorem.

### Theorem 2.13

*Suppose that* $p>1$ *and* $A=(a_{n,k})$ *is a non*-*negative lower triangular matrix*. *If* *A* *is a bounded matrix operator from* $l_{p}(w)$ *into itself*, *then* *A* *is a bounded matrix operator from* $l_{p}(w)$ *into* $c_{p}(w)$ *and*

$$\Vert A \Vert _{p,w,c}\leq p^{*} \Vert A \Vert _{p,w}. $$

### Proof

We have

$$\begin{aligned} \Vert Ax \Vert _{p,w,c}^{p} =&\sum _{n=1}^{\infty}w_{n} \Biggl\vert \frac{1}{n}\sum_{k=1}^{n}\sum _{j=1}^{k} a_{k,j}x_{j} \Biggr\vert ^{p} \\ =&\sum_{n=1}^{\infty}w_{n} \Biggl\vert \sum_{j=1}^{n} (C_{1}A)_{n,j}x_{j} \Biggr\vert ^{p}= \bigl\Vert (C_{1}A)x \bigr\Vert _{p,w}^{p}. \end{aligned}$$

Hence, by Lemma 2.3, we conclude that $\Vert A \Vert _{p,w,c}= \Vert C_{1}A \Vert _{p,w}\le p^{*} \Vert A \Vert _{p,w}$. □

We apply the above theorem to the following two Nörlund and weighted mean matrices.

### Corollary 2.14

[7], Corollary 1.3

*Suppose that* $p>1$ *and* $N_{a}=(a_{n,k})$ *is the Nörlund matrix and* $(a_{n})$ *is a decreasing sequence with* $a_{n}\downarrow\alpha$ *and* $\alpha>0$. *Then*

$$\Vert N_{a} \Vert _{p,w}=p^{*}. $$

### Corollary 2.15

*Suppose that* $p>1$ *and* $N_{a}=(a_{n,k})$ *is the Nörlund matrix and* $(a_{n})$ *is a decreasing sequence with* $a_{n}\downarrow\alpha$ *and* $\alpha>0$. *Then*

$$\Vert N_{a} \Vert _{p,w,c}\le\bigl(p^{*} \bigr)^{2}. $$

### Proof

By applying Theorem 2.13 and Corollary 2.14, we have the desired result. □

### Corollary 2.16

[7], Corollary 1.4

*Suppose that* $p>1$ *and* $M_{a}=(a_{n,k})$ *is the weighted mean matrix and* $(a_{n})$ *is an increasing sequence with* $a_{n}\uparrow\alpha$ *and* $\alpha<\infty$. *Then*

$$\Vert M_{a} \Vert _{p,w}=p^{*}. $$

### Corollary 2.17

*Suppose that* $p>1$ *and* $M_{a}=(a_{n,k})$ *is the weighted mean matrix and* $(a_{n})$ *is an increasing sequence with* $a_{n}\uparrow\alpha$ *and* $\alpha<\infty$. *Then*

$$\Vert M_{a} \Vert _{p,w,c}\le\bigl(p^{*} \bigr)^{2}. $$

### Proof

By using Theorem 2.13 and Corollary 2.16, the proof is clear. □

## The norm of matrix operators from $c_{p}(w)$ into $l_{p}(w)$

In this section, we compute the bounds for the norm of lower triangular matrix operators from $c_{p}(w)$ into $l_{p}(w)$. In particular, when $w_{n}=1$ for all *n*, the bounds for the norm of lower triangular matrix operators from $c_{p}$ into $l_{p}$ are deduced. Moreover, we apply our results for Cesàro, Nörlund and weighted mean matrices.

We begin with a proposition which is needed to prove the main theorem of this section.

### Proposition 3.1

([6], *Proposition *5.1). *Let* $p>1$ *and* $w=(w_{n})$ *be a decreasing sequence with non*-*negative entries*, *and let* $C_{1}$ *be the Cesàro matrix*. *Then we have* $\Vert C_{1} \Vert _{p,w}\leq p^{*}$.

### Theorem 3.2

*Suppose that* $p>1$ *and* $w=(w_{n})$ *is a sequence with non*-*negative entries and* $A=(a_{n,k})$ *is a lower triangular matrix with non*-*negative entries*. *We have*

$$\frac{1}{p^{*}} \Vert A \Vert _{p,w}\leq \Vert A \Vert _{c,p,w}\leq\sup_{n\ge1} \Bigl(n\sup_{1\le k\le n} a_{n,k} \Bigr). $$

*Moreover*, *if the right*-*hand side of the above inequality is finite*, *then* *A* *is a bounded matrix operator from* $c_{p}(w)$ *into* $l_{p}(w)$. *In particular*, *if* $w_{n}=1$ *for all* *n*, *then we have*

$$\frac{1}{p^{*}} \Vert A \Vert _{p}\leq \Vert A \Vert _{c,p}\leq\sup_{n\ge1} \Bigl(n\sup_{1\le k\le n} a_{n,k} \Bigr). $$

### Proof

Suppose that $x\in c_{p}(w)$

$$\begin{aligned} \Vert Ax \Vert _{p,w}^{p} =&\sum _{n=1}^{\infty}w_{n} \Biggl\vert \sum _{k=1}^{n}a_{n,k}x_{k} \Biggr\vert ^{p} \\ \leq&\sum_{n=1}^{\infty}w_{n} \Biggl( \sup_{{1\le k\le n}} a_{n,k}\sum_{k=1}^{n}x_{k} \Biggr)^{p} \\ \leq& \sup_{n\ge1} \Bigl(n\sup_{1\le k\le n} a_{n,k} \Bigr)^{p} \sum_{n=1}^{\infty}w_{n} \Biggl(\frac{1}{n}\sum_{k=1}^{n}x_{k} \Biggr)^{p} \\ =& \sup_{n\ge1} \Bigl(n\sup_{1\le k\le n} a_{n,k} \Bigr)^{p} \Vert x \Vert _{p,w,c}^{p}. \end{aligned}$$

Hence

$$\begin{aligned} \frac{ \Vert Ax \Vert _{p,w}}{ \Vert x \Vert _{p,w,c}}\le\sup_{n\ge1} \Bigl(n\sup _{1\le k\le n} a_{n,k} \Bigr) \end{aligned}$$

and

$$\begin{aligned} \Vert A \Vert _{c,p,w}\leq\sup_{n\ge1} \Bigl(n\sup _{1\le k\le n} a_{n,k} \Bigr). \end{aligned}$$

On the other hand, Proposition 3.1 concludes that $\Vert x \Vert _{p,w,c}= \Vert C_{1}x \Vert _{p,w}\leq p^{*} \Vert x \Vert _{p,w}$, so

$$\begin{aligned} \frac{ \Vert Ax \Vert _{p,w}}{ \Vert x \Vert _{p,w,c}}\ge\frac{1}{p^{*}}\frac{ \Vert Ax \Vert _{p,w}}{ \Vert x \Vert _{p,w}}. \end{aligned}$$

Therefore $\frac{1}{p^{*}} \Vert A \Vert _{p,w}\leq \Vert A \Vert _{c,p,w}$, and the proof is complete. □

### Corollary 3.3

*If* $p>1$, *then the generalized Cesàro matrix* $C_{N}$ *is bounded from* $c_{p}(w)$ *into* $l_{p}(w)$ *and*

$$\Vert C_{N} \Vert _{c,p,w}=1. $$

### Proof

Since

$$\begin{aligned} \sup_{n\ge1} \Bigl(n\sup_{1\le k\le n} a_{n,k} \Bigr)=\sup_{n\ge 1}\frac{n}{n+N-1}=1, \end{aligned}$$

by using Lemma 2.3 and Theorem 3.2, the proof is obvious. □

We apply the above theorem to the following two special cases.

### Corollary 3.4

*Suppose that* $p>1$ *and* $N_{a}=(a_{n,k})$ *is the Nörlund matrix and* $(a_{n})$ *is a decreasing sequence with* $a_{n}\downarrow\alpha$ *and* $\alpha>0$. *Then*

$$1\leq \Vert N_{a} \Vert _{c,p,w}\leq a_{1}\sup _{n\ge1}\frac{n}{A_{n}}. $$

### Proof

By Theorem 3.2 and Corollary 2.14, the proof is clear. □

### Example 3.5

Let $\alpha>0$ and $a_{n}=\alpha+\frac{1}{n^{\alpha+1}}$ for all *n*. The sequence $(a_{n})$ is decreasing and $a_{n}\downarrow\alpha$ and also $a_{1}\sup_{n\ge1}\frac{n}{A_{n}}=1+\frac{1}{\alpha}$. So

$$1\leq \Vert N_{a} \Vert _{c,p,w}\leq1+\frac{1}{\alpha}. $$

Specially $\Vert N_{a} \Vert _{c,p,w}\rightarrow1$, when $\alpha\rightarrow\infty$.

### Corollary 3.6

*Suppose that* $p>1$ *and* $M_{a}=(a_{n,k})$ *is the weighted mean matrix and* $(a_{n})$ *is an increasing sequence with* $a_{n}\uparrow\alpha$ *and* $\alpha<\infty$. *Then*

$$1\leq \Vert M_{a} \Vert _{c,p,w}\leq\sup _{n\ge1}\frac{na_{n}}{A_{n}}. $$

### Proof

By using Theorem 3.2 and Corollary 2.16, the proof is obvious. □

### Example 3.7

Let $a_{n}=1-\frac{1}{(n+1)^{2}}$ for all *n*. The sequence $(a_{n})$ is increasing and $a_{n}\uparrow1$ and also

$$\sup_{n\ge1}\frac{na_{n}}{A_{n}}=\frac{3a_{3}}{A_{3}}\simeq1.091. $$

So

$$1\leq \Vert M_{a} \Vert _{c,p,w}\leq1.091. $$

## Conclusions

In the present study, we considered the problem of finding bounds for the norm of lower triangular matrix operators from $l_{p}(w)$ into $c_{p} (w)$ and from $c_{p}(w)$ into $l_{p} (w)$. Moreover, we computed the norms of certain matrix operators such as Cesàro, Nörlund and weighted mean, and we extended some results of [3, 7].
